# A new plan quality objective function for determining optimal collimator combinations in prostate cancer treatment with stereotactic body radiation therapy using CyberKnife

**DOI:** 10.1371/journal.pone.0208086

**Published:** 2018-11-27

**Authors:** Maria Varnava, Iori Sumida, Hirokazu Mizuno, Hiroya Shiomi, Osamu Suzuki, Yasuo Yoshioka, Kazuhiko Ogawa

**Affiliations:** 1 Department of Radiation Oncology, Osaka University Graduate School of Medicine, Suita, Osaka, Japan; 2 Miyakojima IGRT Clinic, Miyakojima-ku, Osaka, Japan; 3 Department of Carbon Ion Radiotherapy, Osaka University Graduate School of Medicine, Suita, Osaka, Japan; 4 Department of Radiation Oncology, Cancer Institute Hospital of Japanese Foundation for Cancer Research, Koto-ku, Tokyo, Japan; North Shore Long Island Jewish Health System, UNITED STATES

## Abstract

Stereotactic body radiation therapy with CyberKnife for prostate cancer has long treatment times compared with conventional radiotherapy. This arises the need for designing treatment plans with short execution times. We propose an objective function for plan quality evaluation, which was used to determine an optimal combination between small and large collimators based on short treatment times and clinically acceptable dose distributions. Data from 11 prostate cancer patients were used. For each patient, 20 plans were created based on all combinations between one small (⌀ 10–25 mm) and one large (⌀ 35–60 mm) Iris collimator size. The objective function was assigned to each combination as a penalty, such that plans with low penalties were considered superior. This function considered the achievement of dosimetric planning goals, tumor control probability, normal tissue complication probability, relative seriality parameter, and treatment time. Two methods were used to determine the optimal combination. First, we constructed heat maps representing the mean penalty values and standard deviations of the plans created for each collimator combination. The combination giving a plan with the smallest mean penalty and standard deviation was considered optimal. Second, we created two groups of superior plans: group A plans were selected by histogram analysis and group B plans were selected by choosing the plan with the lowest penalty from each patient. In both groups, the most used small and large collimators were assumed to represent the optimal combination. The optimal combinations obtained from the heat maps included the 25 mm as a small collimator, giving small/large collimator sizes of 25/35, 25/40, 25/50, and 25/60 mm. The superior-group analysis indicated that 25/50 mm was the optimal combination. The optimal Iris combination for prostate cancer treatment using CyberKnife was determined to be a collimator size between 25 mm (small) and 50 mm (large).

## Introduction

Stereotactic body radiation therapy (SBRT) using CyberKnife (Accuray Inc., Sunnyvale, CA, USA) is one of the methods of external beam radiotherapy for prostate cancer. CyberKnife, a non-invasive frameless whole-body image-guided radiosurgery system, has various advantages compared with three-dimensional conformal radiation therapy (3D-CRT) and intensity-modulated radiation therapy (IMRT). A 6-MV small linear accelerator is mounted on a computer-controlled robotic arm [1], which unlike 3D-CRT and IMRT, makes irradiation possible from any direction. CyberKnife is also equipped with a real-time imaging system with an orthogonal pair of diagnostic x-ray imaging devices [1], and uses numerous beams with small field sizes that are supported by circular collimators, such as the fixed and Iris variable aperture (Accuray Inc., Sunnyvale, CA, USA) collimators.

The characteristics of CyberKnife allow for accurate delivery of high doses to planning target volumes (PTVs) and for the dose delivered to organs at risk (OARs) to be minimized. In contrast to brachytherapy, the dose delivered to the urethra can also be minimized [2]. Moreover, treatment with CyberKnife is usually hypofractionated, which is thought to be more effective for prostate cancer because the prostate has a lower α/β value—that is, the tumor or normal tissue-specific linear-quadratic parameter that measures a tissue’s sensitivity to fractionation [3]—than its surrounding organs (including the rectum, bladder, and urethra). This indicates that tumor cells are more sensitive to changes in the dose per fraction than normal tissue cells [4].

Despite its clear benefits, CyberKnife has long treatment times per session compared with 3D-CRT and IMRT, which have treatment times of about 10 min [5]. The need for repeated real-time x-ray imaging and the use of numerous beams result in treatment times for prostate cancer ranging between 40 to 90 min [6–8]. The length of the treatment time has a direct effect on patient discomfort, and consequently on setup errors during a session. Therefore, it is of great importance to design treatment plans that have short execution times to reduce unnecessary discomfort in patients with prostate cancer, who may be unable to remain still for long periods of time because of pain, and ensure a more accurate therapy.

Plan quality evaluation is an important feature of radiotherapy, as there is the need to know whether a given treatment plan is deliverable to a given patient. Various radiobiological models are used to evaluate plan quality, including cell survival models (e.g., the linear-quadratic model), tumor control probability (TCP) and normal tissue complication probability (NTCP) models (e.g., the dose–response model), and biologically equivalent doses (e.g., the equivalent uniform dose). Usually, researchers investigate plan quality through dosimetric planning parameters, such as the homogeneity and conformity index [9]. On the other hand, the treatment time is not often used in plan quality evaluation. Recently, a study used a ranking method on a scale from 1 to 4 (from excellent to poor) for multiple criteria parameters, including the treatment time, to evaluate plan quality for spinal robotic radiosurgery [10]. A different method for evaluating plan quality, including the treatment time, is introduced in this study.

We propose an objective function, which combines both dosimetric planning goals and treatment time, to evaluate treatment plans for prostate cancer patients undergoing SBRT using CyberKnife. The objective function was used to determine an optimal combination between small and large collimators based on short treatment times and clinically acceptable dose distributions.

## Materials and methods

This retrospective study was approved by the Institutional Review Board of Osaka University Hospital and written informed consent was obtained from all patients. We included 11 patients with prostate cancer treated between January 2014 and March 2015 for treatment plan simulations. The median age of patients was 66 years at the start of treatment. Treatment plans were developed for each patient and optimized using the CyberKnife treatment planning system, called MultiPlan (version 4.6.1, Accuray Inc., Sunnyvale, CA, USA).

### Treatment planning

The CyberKnife (model G4) installed in our institution is equipped with fixed and Iris variable aperture collimators that can change the irradiated beam size. The latter was selected as the collimator type for planning. We created treatment plans for each patient by using planning computed tomography (CT) images obtained before treatment with a 16-slice multi-detector row CT (Bright Speed Elite; GE Healthcare, Waukesha, WI, USA). The CT images were acquired with a slice thickness of 2.5 mm and with patients in a supine position on a vacuum-formed cushion (Vac-Lok cushion; CIVCO Medical Solutions, Kalona, IA, USA).

The OARs, rectum, bladder, femoral heads, and urethra, were delineated. The urethra, which is normally not visible on CT, was identified by insertion of a Foley catheter before planning CT. Through the Foley catheter, urine was ejected from the bladder and 100 cm^3^ of saline was injected to keep the bladder volume uniform. The prostate was delineated after image registration between the planning CT and magnetic resonance images, which were acquired prior to the planning CT images. The clinical target volume (CTV) was defined as the sum of the prostate and proximal seminal vesicles, plus 3 mm or 1 mm in the posterior direction (to avoid the rectum). The PTV was defined as the CTV plus 2 mm. The targeting beam was set at 5 mm inside the PTV boundary. A dosage of 40 Gy was applied in five fractions to cover 95% of the PTV.

The dosimetric planning goals are shown in Table 1, and correspond to hypofractionated radiotherapy for low- and intermediate-risk prostate cancer using robot-tracking SBRT in phase I/II clinical trials. These goals are based on the planning goals for high-dose-rate (HDR) brachytherapy. Because SBRT with CyberKnife is comparable to HDR brachytherapy in delivering high doses to the tumor, by choosing planning goals based on HDR brachytherapy, high-conformal tumor-volume coverage and intraprostatic dosimetry control are preserved [11]. The goal for the treatment time was set as ≤30 min, including the 5-min patient setup time and the 1-min image time interval for continuous image guidance during treatment. We created 20 plans per patient. The difference was the combination of the collimators used, with plans created using all combinations between one small (⌀ = 10, 12.5, 15, 20, and 25 mm) and one large (⌀ = 35, 40, 50, and 60 mm) collimator size. In total, 220 plans were created by sequential optimization in MultiPlan. The optimization script was kept static for all patients. Dose calculations were performed using the ray-tracing algorithm with tissue heterogeneity corrections. The voxel size used for dose calculations was 1 × 1 × 2.5 mm^3^.

**Table 1:** Dosimetric planning goals used for designing treatment plans.

Organ at Risk	Planning Goal^a^
Rectum	D2cc < 35 Gy, D5cc < 30 Gy, V50% < 40%
Bladder	D10cc < 35 Gy, V50% < 35 cc, V100% < 5 cc
Femoral head (left and right)	V40% < 5%
Urethra	D10% < 50 Gy, D30% < 45 Gy

^a^Vxx: volume of OAR receiving xx% of dose, Dxx: dose incident on xx% or xx cc OAR volume

### Evaluation of plan quality

To determine the optimal collimator combination, we evaluated the quality of all 220 plans. An objective function was designed and assigned to each plan as a penalty. This function takes into consideration the generalized equivalent uniform dose (gEUD), and the gEUD derived TCP and NTCP. Niemierko defined the gEUD as the dose causing the same biological effect if uniformly distributed throughout the entire tumor or OAR volume as the actual non-uniform dose distribution [3, 12, 13]:

$$gEUD={\left(\sum_{i=1}\left(v_{i}{EQD}_{i}^{a}\right)\right)}^{\frac{1}{a}},$$ (1)

where *v*_*i*_ is the partial volume in the *i*^th^ voxel receiving dose *D*_*i*_ in Gy, and *a* is a parameter specific to the tumor or normal tissue that describes the dose–volume effect. Both *v*_*i*_ and *a* are unitless. The EQD represents the biologically equivalent physical dose of 2 Gy given by [3]:

$${EQD}_{i}=D_{i}∙\frac{\left(\frac{\alpha }{\beta }+\frac{D}{n_{f}}\right)}{\left(\frac{\alpha }{\beta }+2\right)}.$$ (2)

In this equation, *n*_*f*_ is the number of fractions and *D*/*n*_*f*_ is the dose per fraction of the treatment course. The EQD is used in the calculation of the gEUD instead of the actual dose to enable comparison with different fractionation regimes from the conventional ones that use 2 Gy per fraction, because many tissue toxicities are given with a total dose considering conventional fractionation.

Based on the gEUD, the TCP and NTCP can be defined as follows [12–14]:

$$TCP=\frac{1}{1+{\left(\frac{{TCD}_{50}}{gEUD}\right)}^{4\gamma 50}},$$ (3)

$$NTCP=\frac{1}{1+{\left(\frac{{TD}_{50}}{gEUD}\right)}^{4\gamma 50}}.$$ (4)

The parameter *γ*50 is unitless, specific to tumor or normal tissue, and describes the slope of the dose–response curve. *TCD*_50_ refers to the dose needed to control 50% of the tumor when the tumor is homogeneously irradiated, while *TD*_50_ refers to the tolerance dose that would produce a 50% complication rate at a specific time interval (e.g., 5 years) [14, 15].

The objective function we designed penalized overdosing of critical structures and long treatment times, and is given by the equation:

$$Penalty=\left[\sum_{i}^{N}\left(\frac{X_{plan}^{i}-X_{goal}^{i}}{X_{goal}^{i}}\right)\right]∙[1-TCP∙\prod_{k}^{M}\left(1-s_{k}∙NTCP\right)]+\frac{t_{plan}-t_{goal}}{t_{goal}},$$ (5)

where *X*^*i*^_*goal*_/*X*^*i*^_*plan*_ is the *i*^th^ dose−volume index from the planning goals/the optimized plan, *s*_*k*_ is the relative seriality parameter of the *k*^th^ OAR, and *t*_*goal*_/*t*_*plan*_ is the treatment time from the planning goals (30 min)/the optimized plan. The parameters *N* and *M* represent the number of the dose−volume indices and OARs, respectively.

The objective function comprises three distinct parts. The first part corresponds to the level of achievement of the dosimetric planning goals. In case the value of the dose−volume index from the optimized plan was less than the value from the planning goal, the *X*^*i*^_*plan*_−*X*^*i*^_*goal*_ difference was set equal to zero. The second one corresponds to the probability that there is no tumor control, but there are complications in normal tissues [16]. This probability was modified to account for the radiosensitivity of the OARs based on their structure, using the relative seriality parameter [17, 18]. These data and the parameters necessary to calculate the TCP (Eq 3) and NTCP (Eq 4) are shown in Table 2. From here onwards, the product of the first and second part of the objective function will be referred to as the dosimetric penalty. The third one corresponds to the achievement level of the time planning goal. Similar to the first part, if the treatment time from the optimized plan was <30 min, the *t*_*plan*_−*t*_*goal*_ difference was set equal to zero. In case all planning goals were achieved, the penalty became zero: thus, the lower the penalty value, the better the quality (i.e., the greater the superiority) of the treatment plan.

**Table 2:** Parameters used for TCP, NTCP, and penalty evaluation.

	*a*	*TD*_50_/*TCD*_50_	*γ*50	*α*/*β*	*s*	Clinical endpoint	Study^b^
**Prostate**	−13	67.5	2.2	1.5	-	-	[12, 19, 20]
**Rectum**	8.33	80	4	3	0.75	severe proctitis/necrosis/ stenosis/fistula	[12, 19]
**Bladder**	2	80	4	3	1.3	symptomatic bladder contracture and volume loss	[12, 19]
**Femoral heads**	4	65	4	0.85	1	necrosis	[12, 19, 21]
**Urethra**^**a**^	19	68	4	3	1	clinical stricture/perforation	[13, 22, 23]

^a^It was assumed that the parameters of urethra are the same as esophagus because they have the same anatomical structure; both are considered serial structures

^b^reference to study

Before using the objective function, we assessed whether it sufficiently penalizes excess irradiation to the OARs and long treatment times by means of Spearman’s rank-order correlation analysis. Correlations between the penalty values and various parameters, including the dose−volume indices from Table 1, PTV D95%, TCP, NTCPs of all OARs, treatment time, monitor units (MUs), and the homogeneity (HI) and conformity (CI) indices, were investigated. We hypothesized that penalty values would be negatively correlated with the TCP and PTV D95%, while they would be positively correlated with the rest of the parameters.

### Optimal collimator combination

We compared the outcomes of two methods to determine the optimal collimator combination.

First, we constructed two heat maps [24] of all 220 plans that showed the mean penalty values and standard deviations created by each combination. The combination giving a plan with a low mean penalty and small standard deviation was considered optimal.

In the second method, however, we only considered superior plans and classified them as group A and group B plans. Group A comprised plans with small penalty values (superior plans) identified from a histogram of the penalty values of all 220 treatment plans. Before plotting the histogram, the appropriate bin number, *k*, for presenting the penalty distribution was estimated using Doane’s formula for non-normal data as follows [25]:

$$k=1+{log}_{2}n+{log}_{2}\left(1+\frac{\sqrt{b_{1}}}{\sigma \sqrt{b_{1}}}\right),$$ (6)

where *n* is the total number of penalty values, the parameter $\sqrt{b_{1}}$ is a measurement of skewness equal to $\left[\sum_{i=1}^{N}{\left(x_{i}-\bar{x}\right)}^{3}\right]/{\left[\sum_{i=1}^{N}{\left(x_{i}-\bar{x}\right)}^{2}\right]}^{3/2}$, *x*_*i*_ is the penalty of the *i*^th^ collimator combination, and $\sigma \sqrt{b_{1}}$ is the standard deviation of $\sqrt{b_{1}}$, which depends only on the sample size and can be calculated from $\sqrt{6∙(n-2)/\left[\left(n+1)∙(n+3\right)\right]}$.

Group B was formed by considering the plans with the smallest penalty values per patient, and was used to identify the collimator sizes used most often in the best-quality plans, based on the objective function (Eq 5); only 11 plans were chosen.

For the superior plans in groups A and B, we calculated the frequency of each collimator size used, and assumed that the highest-frequency small and large sizes would give the optimal combinations. These sizes formed combinations that produced the majority of superior plans, therefore a combination between the highest-frequency small and large sizes would most likely produce a superior plan.

The optimal combinations obtained from the heat map and superior-group analyses were compared with the other combinations used in this study to investigate whether these optimal combinations produced plans with significantly lower penalties using a Wilcoxon signed-rank test. A *p*-value <0.05 was considered statistically significant. To account for multiple testing, the Bonferroni-Holm correction was used to adjust the significance level [26]. After evaluating the outcomes of the heat map and superior-group analyses, we determined the optimal collimator combination for prostate cancer treatment with SBRT.

## Results

### Evaluation of plan quality

The feasibility of the objective function was evaluated through correlation analyses. In total, 12 out of the 21 parameters investigated had significant correlations with the penalty values. Table 3 shows the correlation coefficients (*r*) and *p*-values of all parameters. The treatment time and MUs showed very strong (*r* = 0.891) and strong (*r* = 0.770) correlations, respectively. A moderate correlation was observed for the rectum NTCP (*r* = -0.524). Significant weak and very weak correlations were also found for the bladder D10cc (*r* = 0.350), right femoral head NTCP (*r* = 0.346), bladder V50% (*r* = 0.300), TCP (*r* = 0.275), right femoral head V40% (*r* = 0.262), PTV D95% (*r* = -0.208), left femoral head NTCP (*r* = 0.206), urethra D10% (*r* = 0.191), and bladder NTCP (*r* = 0.190). Non-expected correlation signs were observed for the rectum NTCP and TCP, which had a negative and positive correlation, respectively. Although the TCP had a positive correlation, PTV D95% had a negative correlation, as expected. At least one parameter from each organ of interest, except the rectum, as well as the treatment time and MUs, had the expected significant correlations. From this, we deduced that the objective function can sufficiently penalize overdose to the OARs and long treatment times.

**Table 3:** Correlations between penalty values and treatment planning parameters.

	Parameter	Spearman’s correlation coefficient (*r*)	*p*-value^a^	Bonferroni-Holm significance level^b^
	Treatment time	0.891*	<0.001	0.002
	MU	0.770*	<0.001	0.003
**PTV**	D95%	-0.208*	0.002	0.004
	CI	-0.153	0.023	0.006
	HI	0.055	0.420	0.050
	TCP	0.265*	<0.001	0.003
**Rectum**	D2cc	-0.091	0.181	0.010
	D5cc	0.067	0.321	0.017
	V50%	-0.076	0.264	0.013
	NTCP	-0.525*	<0.001	0.003
**Bladder**	D10cc	0.350*	<0.001	0.003
	V50%	0.300*	<0.001	0.003
	V100%	-0.108	0.110	0.007
	NTCP	0.190*	0.005	0.005
**Urethra**	D10%	0.191*	0.004	0.005
	D30%	-0.096	0.158	0.008
	NTCP	0.130	0.053	0.006
**Left femoral head**	V40%	0.064	0.372	0.025
	NTCP	0.205*	0.004	0.004
**Right femoral head**	V40%	0.262*	<0.001	0.004
	NTCP	0.340*	<0.001	0.003

*statistically significant correlations after Bonferroni-Holm correction

^a^*p*-values obtained from the Spearman’s rank-order correlation analysis

^b^the corrected significance level after using the Bonferroni-Holm method

**Fig 1:**
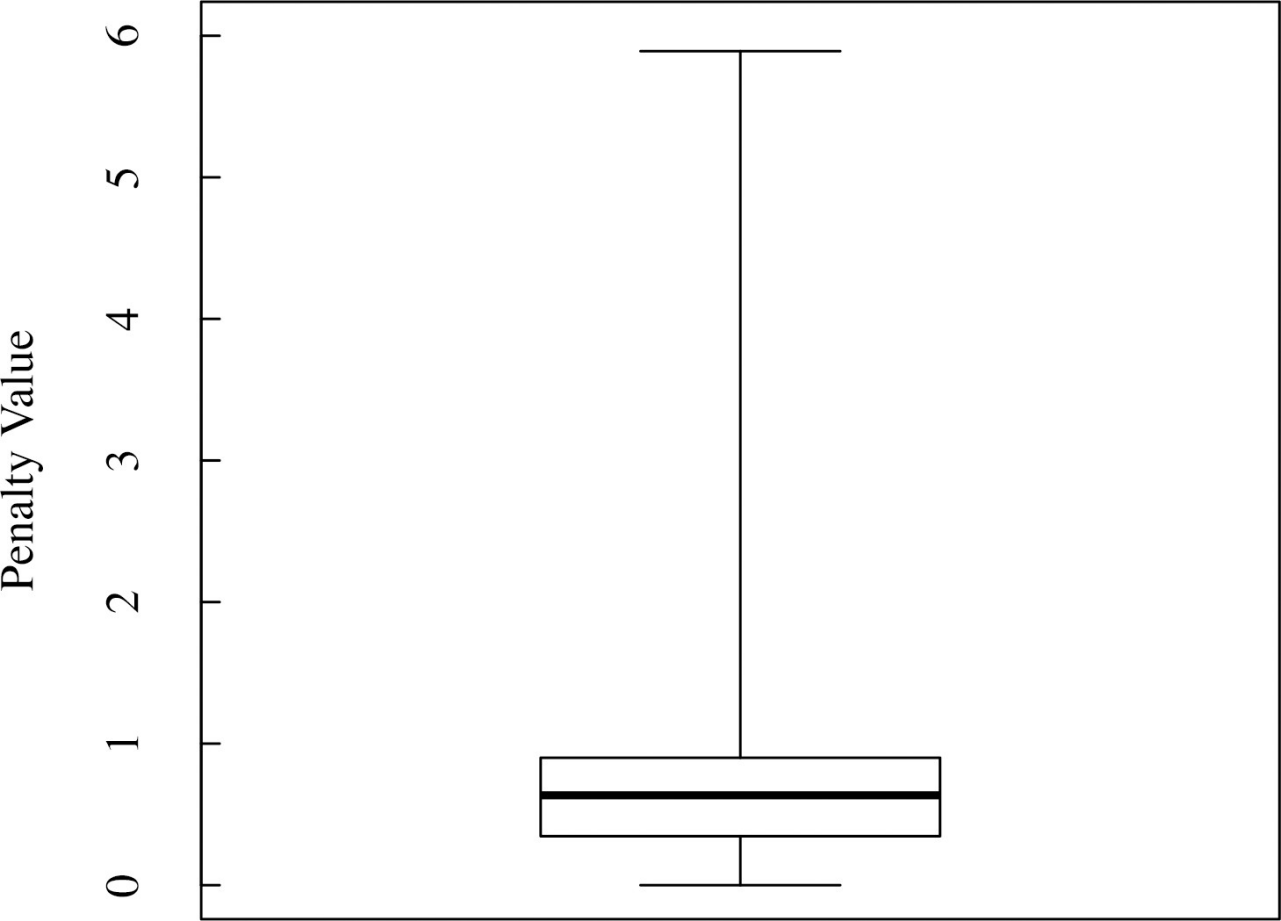
Distribution of all penalty values. The box plot represents the first quartile, median and third quartile of the penalties. The whiskers correspond to the minimum and maximum penalty values.

The 220 plans had penalty values ranging from 0 to 5.89 (median, 0.63), as obtained from the objective function (Eq 5), and treatment times ranging from 24 to 90 min (mean, 48 min). A box plot of the penalty values is shown in Fig 1, with a positive skew evidenced by more plans with small penalty values than large values. Dosimetric and time planning goals were achieved in 79 and 12 treatment plans, respectively. However, only one plan achieved both dosimetric and time planning goals.

### Optimal collimator combination

**Fig 2:**
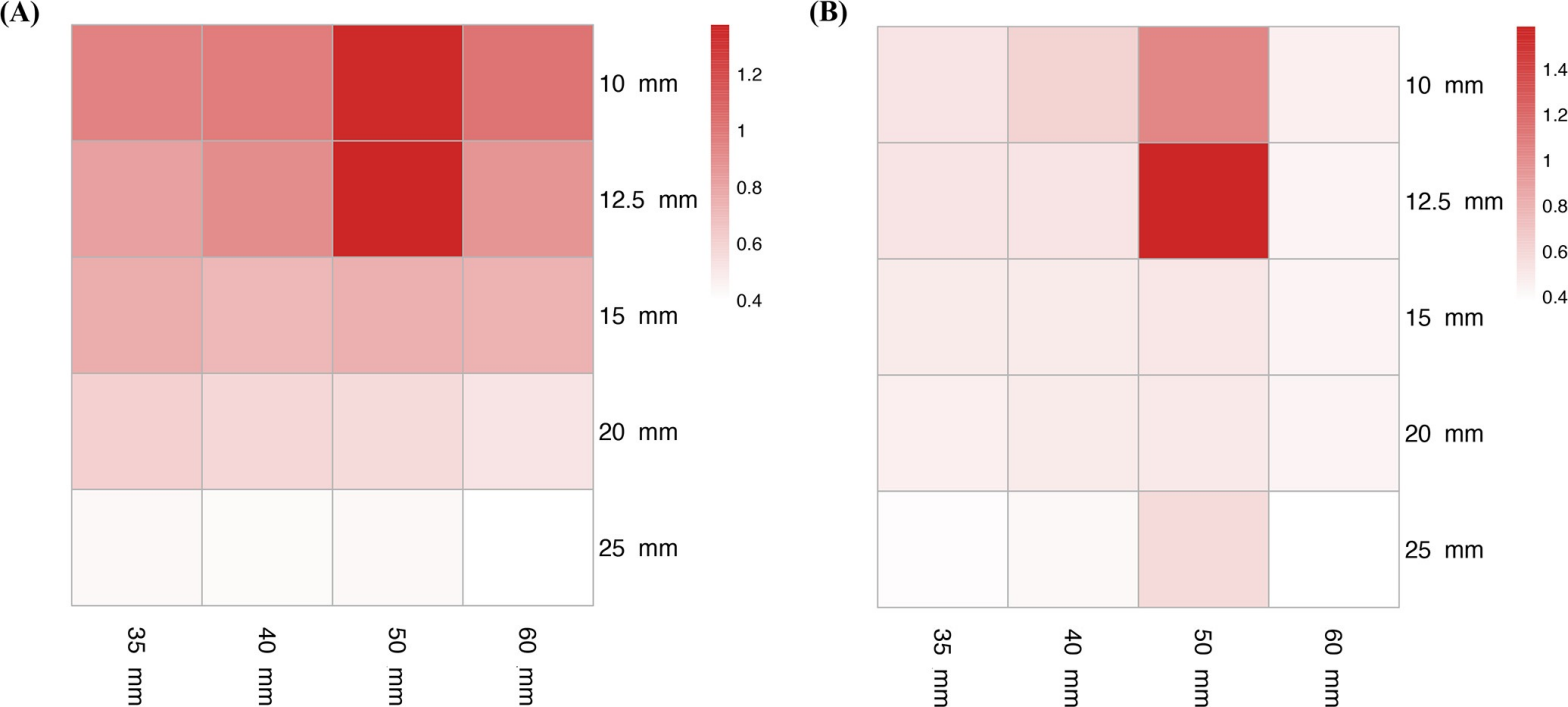
Heat maps of means and standard deviations of plan penalties. The heat maps show the means (A) and standard deviations (B) of the penalties of all collimator combinations between small and large sizes. Rows and columns correspond to small and large sizes, respectively. White and red indicate the minimum and maximum values per heat map, respectively.

Heat maps of the mean penalty values and standard deviations of the plans created by each of the 20 collimator combinations can be found in Fig 2. As can be seen from the heat maps, combinations with the 25 mm collimator size produced plans with the lowest mean penalty values and standard deviations, and the 25/60 mm (small/large collimator size) combination had the smallest standard deviation. The 25/50 mm had a larger standard deviation than the other three combinations, but a similar mean penalty to the 25/35 and 25/40 mm. Thus, we deduced that the 25/35, 25/40, 25/50, and 25/60 mm combinations could be optimal, whereas the 10/50 and 12.5/50 mm combinations, which had the largest mean penalty values and standard deviations, were unlikely to produce clinically acceptable plans.

**Fig 3:**
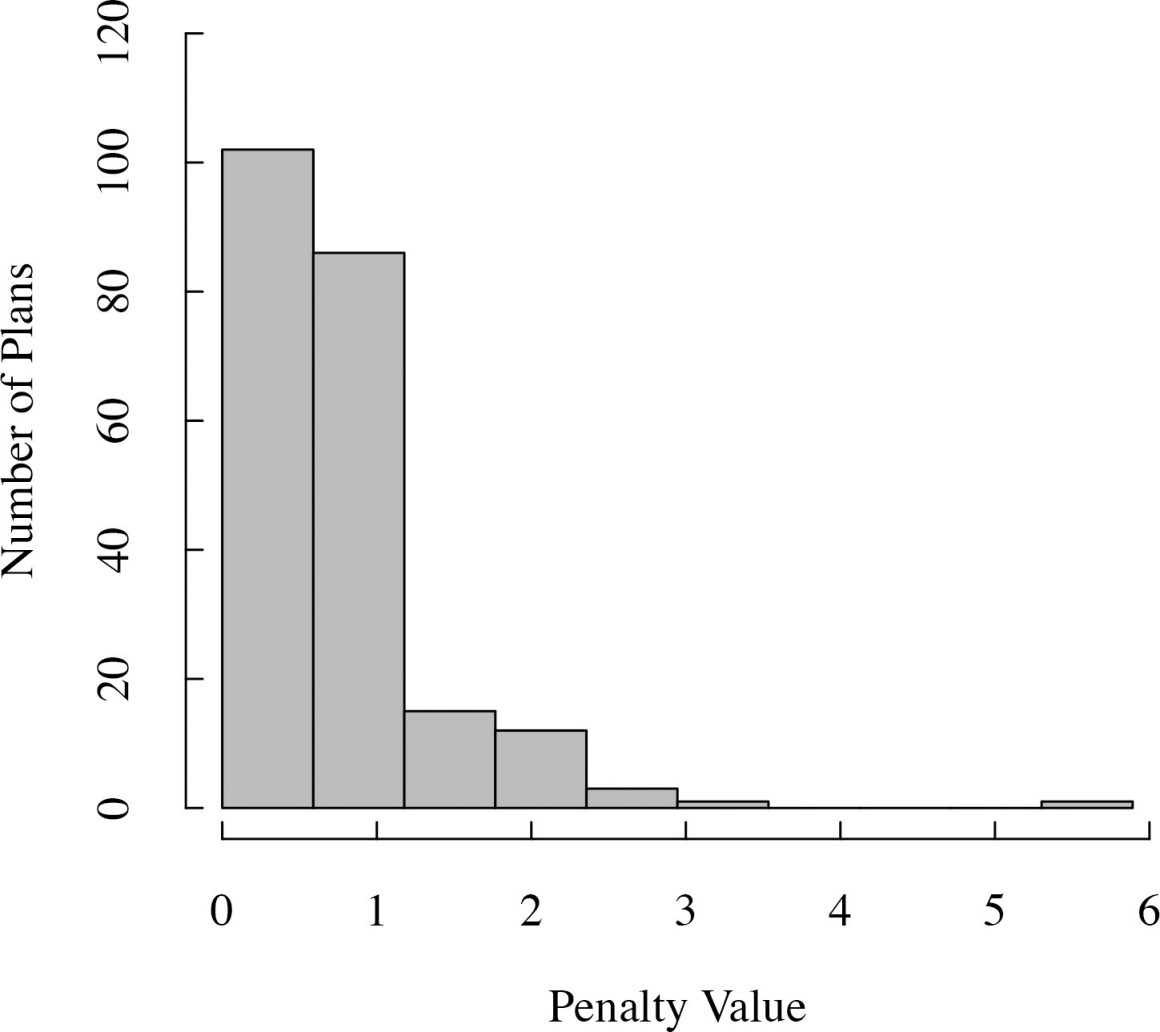
Histogram of the penalty values of treatment plans (*n* = 220).

Fig 3 shows a histogram of the penalty values of all 220 plans. We first confirmed by box plot (Fig 1) that the penalty distribution of the plans was not normal, then by using Doane’s formula (Eq 6) we calculated the bin number to be 10. Based on the penalty distribution, the 102 plans comprising the first bin of the histogram were included in group A. The superior plans most frequently had a small collimator size of 25 mm and large collimator sizes of 35 and 50 mm (Table 4). A similar result was shown for group B with 11 plans (Table 4). The most frequent small and large collimator sizes were the 25 and 50 mm, respectively. The mean treatment time for the plans in group A was 39 ± 8 min (mean ± standard deviation), whereas the mean treatment time for the plans in group B was 35 ± 8 min.

**Table 4:** Frequency of collimator sizes used for the superior plans in groups A and B.

Group	Measure	Small size					Large size			
**A**	Collimator size [mm]	10	12.5	15	20	25	35	40	50	60
	Number	10	10	15	31	36	27	23	27	25
	Percentage [%]	9.80	9.80	14.71	30.39	35.29	26.47	22.55	26.47	24.51
**B**	Collimator size [mm]	10	12.5	15	20	25	35	40	50	60
	Number	0	0	1	1	9	0	3	5	3
	Percentage [%]	0	0	9.09	9.09	81.82	0	27.27	45.45	27.27

The possible optimal combinations, as obtained from the heat map and superior-group analyses, were the 25/35, 25/40, 25/50, and 25/60 mm. Comparisons between the penalties produced by each possible optimal combination and the remaining nineteen collimator combinations showed significant differences between the 25/35 mm and five combinations, the 25/40 mm and six combinations, the 25/50 mm and six combinations, and the 25/60 mm and seven combinations. In most cases, the significant differences revealed lower penalty values for the plans created using the 25/35, 25/40, 25/50, and 25/60 mm combinations. Significant differences were also observed between the possible optimal combinations. The 25/40 and 25/60 mm combinations produced plans with lower penalty values than the 25/35 mm combination, while the 25/50 mm combination produced plans with higher penalty values.

The plans created using the possible optimal collimator combinations (25/35, 25/40, 25/50, and the 25/60 mm) ranked between the second and sixth, first and fifth, first and nineteenth, and first and eleventh best plans in all patients, respectively. In general, the possible optimal combinations produced good-quality plans. However, for one patient, the 25/50 mm combination produced a plan with the second largest penalty. By excluding this plan from the statistical analysis, the penalties of the 25/50 mm plans became significantly lower than the penalties of the 25/35 mm plans, significant differences were found between the 25/50 mm and eight collimator combinations, while the plans created with the 25/50 mm ranked between the first and seventh best plans.

## Discussion

We introduced a new objective function for evaluating plan quality in prostate cancer treatment with CyberKnife and used it to determine an optimal collimator combination. To be considered optimal, the combination was required to produce a treatment plan with the shortest treatment time, while retaining clinically acceptable dose distributions. We used the Iris collimator because it could produce plans with shorter treatment times than fixed collimators, in which case extra time would be required to exchange collimators physically or to perform multiple traversals with a robotic manipulator, one traversal for each fixed collimator used [27].

The objective function (Eq 5) combined the success of the dosimetric planning goals, the Niemierko-based TCP and NTCP, the relative seriality parameter, and the achievement of the treatment time planning goal. Although the Niemierko-based parameters and relative seriality parameter were derived from different radiobiological models, they were both used to create the objective function. By combining them, we could account for the severity of a complication to an OAR based on its response to a specific dosage. As is known, the dose–response relation of an organ depends on its architecture, which can be characterized by the relative seriality parameter (*s*) [18]. Organs can be serial, parallel or a mixture of both. In this model, the response of serial organs (*s* ≈ 1), such as the spinal cord, esophagus or urethra, is affected by the maximum dose, while the response of parallel organs (*s* ≈ 0), such as the lungs or liver, is determined by the mean dose delivered [17, 18].

Our objective function could sufficiently penalize overdosing to critical structures and long treatment times and was deemed appropriate to use. Significant correlations were observed for the treatment time, MUs, and at least one parameter of each organ of interest. Almost all significant correlations had the anticipated sign, except the correlations for the TCP and rectum NTCP. Even though the TCP had a positive correlation, PTV D95%, which is also a parameter of the target, had the expected negative correlation. Both TCP and rectum NTCP had values with range less than 1% (TCP: 98.986%−99.960%; rectum NTCP: 0.001%−0.889%). The rectum NTCP values were concentrated at lower percentages, while the TCP values were more evenly distributed. Because of this, it can be assumed that the penalty is more susceptible to changes in parameters other than the rectum NTCP and TCP. The presence of unexpected correlation coefficient signs and the lack of strong correlations information between the penalty and dosimetric parameters indicate that the incorporation of multiple factors into a single penalty value provides different information about treatment plans than the individual dosimetric parameters. However, at least one parameter from almost every organ of interest, the treatment time, and MUs had the anticipated correlations, implying that the objective function can be used for evaluating plans.

Even though the objective function was regarded as satisfactory in evaluating plan quality, there were some cases for which the treatment time affected the objective function. For example, in one case, both the 10/60 and 25/40 mm combinations produced plans with similar dosimetric penalties (penalties evaluated without considering the treatment time); these plans ranked eighth and ninth best, respectively. Although the dosimetric penalties of the plans were similar, the treatment time associated with the 10/60 mm combination (54 min) was double that associated with the 25/40 mm combination (27 min). Thus, when the treatment time was considered in the penalty evaluation, the 25/40 mm combination became the optimal combination for this patient, producing the plan with the smallest penalty, while the ranking of the 10/60 mm combination decreased further. In the future, either a weight factor should be added to the time term or the objective function should be modified. An example would be to define the objective function as the product of the three terms, instead of adding the time term to the dosimetric penalty:

$$Penalty=\left[\sum_{i}^{N}\left(\frac{X_{plan}^{i}-X_{goal}^{i}}{X_{goal}^{i}}\right)\right]∙[1-TCP∙\prod_{k}^{M}\left(1-s_{k}∙NTCP\right)]∙\left(\frac{t_{plan}-t_{goal}}{t_{goal}}\right).$$ (7)

However, this was deemed inappropriate because most plans in which all dosimetric planning goals were achieved had a high-penalty time term due to long treatment times, and plans that met the time goal had high dosimetric penalties. In either case, the objective function would become zero, indicating optimal plans and collimator combinations. However, some of the resulting optimal plans would have long treatment times or large radiation exposures to the OARs. Long treatment times require patients to lie still for long periods of time, increasing their discomfort and the possibility they will move causing excess radiation to reach normal tissue [8]. Moreover, allowing long treatment times or excess irradiation to the OARs would conflict with our stated aims, so we did not use an objective function in which all terms were multiplied. We suggest that a weight factor should be assigned to the time term to balance its effect with the effects from the other terms. The weight factor should correspond either to the patients’ condition based on their physicians’ judgment or to the time difference between the time planning goal (30 min) for treatment with CyberKnife and the time obtained from the optimized plan.

A different objective function has been used in one study to evaluate treatment plans for head-and-neck cancer treated with intensity-modulated proton therapy [28]. The function, called plan-score, was defined as the weighted sum of the differences between the obtained value and the desired value for each planning goal. The weights used in the plan-score were determined according to the priority of the goals as defined by the user in the “wish-list” of the treatment planning system. The plan-score is similar to our penalty. Both objective functions consider the deviation of the obtained values from the dosimetric planning goals. However, the plan-score did not include any radiobiological parameters, like the NTCP to consider for organ complication probabilities, or the treatment time, which is a parameter that should be included in plan evaluation, particularly for cases that have long treatment times per session. Another study used a different method to evaluate treatment plans for spinal robotic radiosurgery [10]. Multiple criteria parameters of the plans, including the treatment time, were mathematically rated from a scale of 1−4. Contrary to a ranking method, our objective function gives the treatment time a greater weight in plan quality evaluation, and combines it with the dosimetric planning goals. The result is a single penalty value that can be used to compare the quality of different treatment plans.

Using the proposed objective function, we investigated the optimal combination between small and large collimator sizes. Previous reports have suggested that large collimators tend to maximize dose uniformity within the target volume and minimize the treatment time, by minimizing the total monitor units and the number of beams, whereas small collimators achieve high-dose conformity and steep dose gradients around the target volume [27, 29]. Another treatment planning study reported that the combination of two collimators can reduce the total monitor units by 31% compared with a single collimator [30]. Therefore, we proposed using a collimator combination between one small and one large collimator size when designing treatment plans.

A few studies recommended the use of multiple collimator sizes [27, 31]. Echner *et al*. compared plans created using one fixed, three fixed, and 12 Iris collimators [27]. Their results showed that using multiple collimator sizes yielded improvements in plan quality. However, much of the plan quality could be achieved using three collimator sizes with a smaller incremental increase offered by using all 12 collimator sizes. Fuller *et al*. also concluded that using multiple Iris collimator sizes can produce treatment plans with better quality that can be delivered more efficiently than plans created using one or two fixed collimator sizes [31]. As an additional test, we created treatment plans using 10 Iris collimator sizes (10−60 mm) for all 11 prostate cancer patients and compared them with the possible optimal combinations we found. There were no significant differences between the dosimetric penalty when using 10 collimators and that when using the 25/35, 25/40, 25/50, and 25/60 mm combinations. These findings are in contradiction with the results of Fuller *et al*., who showed that multiple collimator sizes improve the quality of the plans. However our results are consistent with those of Echner *et al*., who deduced that a small number of collimators can achieve a satisfactory quality that does not differ much from the quality achieved by multiple collimators. Furthermore, the mean treatment time of the 10-collimator plans was 59 ± 13 min. Compared with the mean time of the four possible optimal collimators, the mean time of the 10-collimator plans was about 20 min longer. We believe that a treatment time difference of 20 min is too large to ignore. It is important to take into consideration not only the fulfilment of the dosimetric planning goals, but also the length of the treatment time. As increasing the number of Iris collimators generally yields improved dose−volume indices and longer treatment times, the number of collimators should be adjusted such that a balance between the dose−volume indices and treatment time is achieved. A future study could investigate the appropriate number and sizes of Iris collimators required to optimize plan quality based on the present findings.

The penalty values of all 220 plans, as obtained from the objective function, were spread over a wide range (Fig 1). This was because a few plans had very high dosimetric penalties resulting from a combination of a high penalty for the dose−volume indices due to excess radiation to the OARs, and a high-penalty time term due to long treatment times. As mentioned previously, usually, plans with achieved dosimetric planning goals did not satisfy the treatment time planning goal, while plans that met the time planning goal had a non-zero dosimetric penalty. Only one plan achieved both the dosimetric and time planning goals showing the difficulty to produce a plan with all planning goals achieved. Hence, it is important to achieve a balance between them.

Four possible optimal collimator combinations were found from the heat maps, with all including 25 mm as the small size (smallest mean penalty values). The 25/60 mm combination had the smallest standard deviation (0.40 ± 0.38), while the 25/50 mm had the largest standard deviation (0.44 ± 0.58). Although the standard deviation of the 25/50 mm combination was larger than that of some combinations without the 25 mm size, we still considered it a possible optimal combination. As was mentioned in the Results, the 25/50 mm produced a plan with the second largest penalty value for one patient. This lead to the quite large deviation of the 25/50 mm combination. If we exclude this penalty value from the calculation, the 25/50 mm combination will have the smallest mean penalty and a standard deviation that will approximate that of the 25/40 mm combination (25/50 mm: 0.31 ± 0.42; 25/40 mm: 0.42 ± 0.42). This suggests that 25/50 mm could be an optimal combination, but might not be suitable in all cases. The heat maps also revealed relatively high standard deviations for the penalty means, indicating that the penalties for each combination were spread among patients, probably due to anatomical differences; for example, the sizes of organs of interest, the arrangement of the OARs surrounding the prostate and their distance from the target. The geometric distribution of the OARs might have affected the dose distribution and the time needed to irradiate the PTV uniformly while avoiding the OARs, resulting in different treatment times and different doses being delivered to the OARs. If a patient has a small prostate and the OARs are distanced from it, dosimetric planning goals are easier to achieve than when a patient has a large prostate whose circumference interacts closely with the OARs [6]. Therefore, penalties produced from the same collimator combination will vary among patients.

Among the 102 superior plans in group A and the 11 superior plans in group B, the 25 and 50 mm sizes were the most often used small and large collimators, respectively. Although the 35 mm size had the same frequency as the 50 mm size in group A, none of the superior plans in group B were created using the 35 mm. Wilcoxon signed-rank tests showed that the 25/50 mm combination resulted in plans with significantly lower penalty values compared with the 25/35 mm combination, when the irregular 25/50 mm plan with the high penalty was not included in the analysis. Besides the 25/50 mm plans, the 25/40 and 25/60 mm plans also had better quality compared with the 25/35 mm plans. Therefore, the 25/50 mm combination was selected as optimal from the superior-group analysis. This optimal combination is consistent with the results from the heat maps. Furthermore, from the analysis of group B, we showed that except the 35 mm, the 10 mm size was also not used, indicating that these collimator sizes are unlikely to form combinations that produce plans with the smallest penalty values. The 25/35 mm collimator combination, however, might give a good-quality plan because it always ranked between the second and sixth best plans in all patients.

The possible optimal collimator combinations deduced from the heat maps and the superior-group analysis, namely the 25/35, 25/40, 25/50, and 25/60 mm combinations, produced plans with mean treatment times of 39 ± 11, 39 ± 11, 36 ± 10, and 37 ± 7 min, respectively. One study has reported that the time for prostate cancer treatment with CyberKnife was about 90 min per session [7]. However, other researchers have shown mean treatment times of 43 min, excluding the time needed for patient setup [6], and 40–65 min [8]. Each of these studies required longer mean treatment times than plans based on possible optimal combinations in this study. Nevertheless, we believe that it is possible for the mean treatment times obtained in this study to be further reduced, not least because we did not use any tools for reducing the treatment time during plan optimization with MultiPlan. These tools include reduction of time, beams, nodes (robotic arm positions), and monitor units. Planning goals can still be met, and the integrity of the plan can be preserved, even after using the above tools to decrease the treatment time [32]. Studies that used the above reduction tools when creating treatment plans for prostate cancer patients with multiple Iris collimators found mean treatment times of 23.9 min [31] and 34.0 ± 5.0 min [33]. The first mean time (23.9 min) is shorter than the times of our possible optimal collimators, while the latter one (34.0 min) is comparable to the mean time of the plans created using the 25/50 mm combination. Compared with the mean treatment time of our 10-collimator plans (59 min), both reported times are shorter. This is expected as no time reduction tools were used in our study. From these, it can be deduced that the treatment times of our plans can be reduced if the time reduction tools are utilized during planning. Moreover, MultiPlan was recently upgraded to version 5.2.1 and can now produce plans with treatment times of up to 10 min shorter than for the same plans produced by version 4.6.1. Therefore, by using MultiPlan version 5.2.1 with the time reduction tools, it should be possible to produce plans with even shorter treatment times for the four possible optimal collimator combinations that we identified.

Among the 11 optimal plans from group B, 40 mm was used three times for patients with small prostate volumes (<20.80 cm^3^) and PTVs (<43.33 cm^3^). Although the 60 mm size was expected to form optimal combinations for patients with large PTVs, that was not the case. The treatment time was short when using 60 mm as the large size and decreased the time term of the objective function; however, the dosimetric penalty had a relatively high value, resulting in a large overall penalty. Because the 60 mm size is large, it is difficult to avoid the OARs, which will consequently receive higher doses when compared with the 35, 40, and 50 mm collimator sizes.

**Fig 4:**
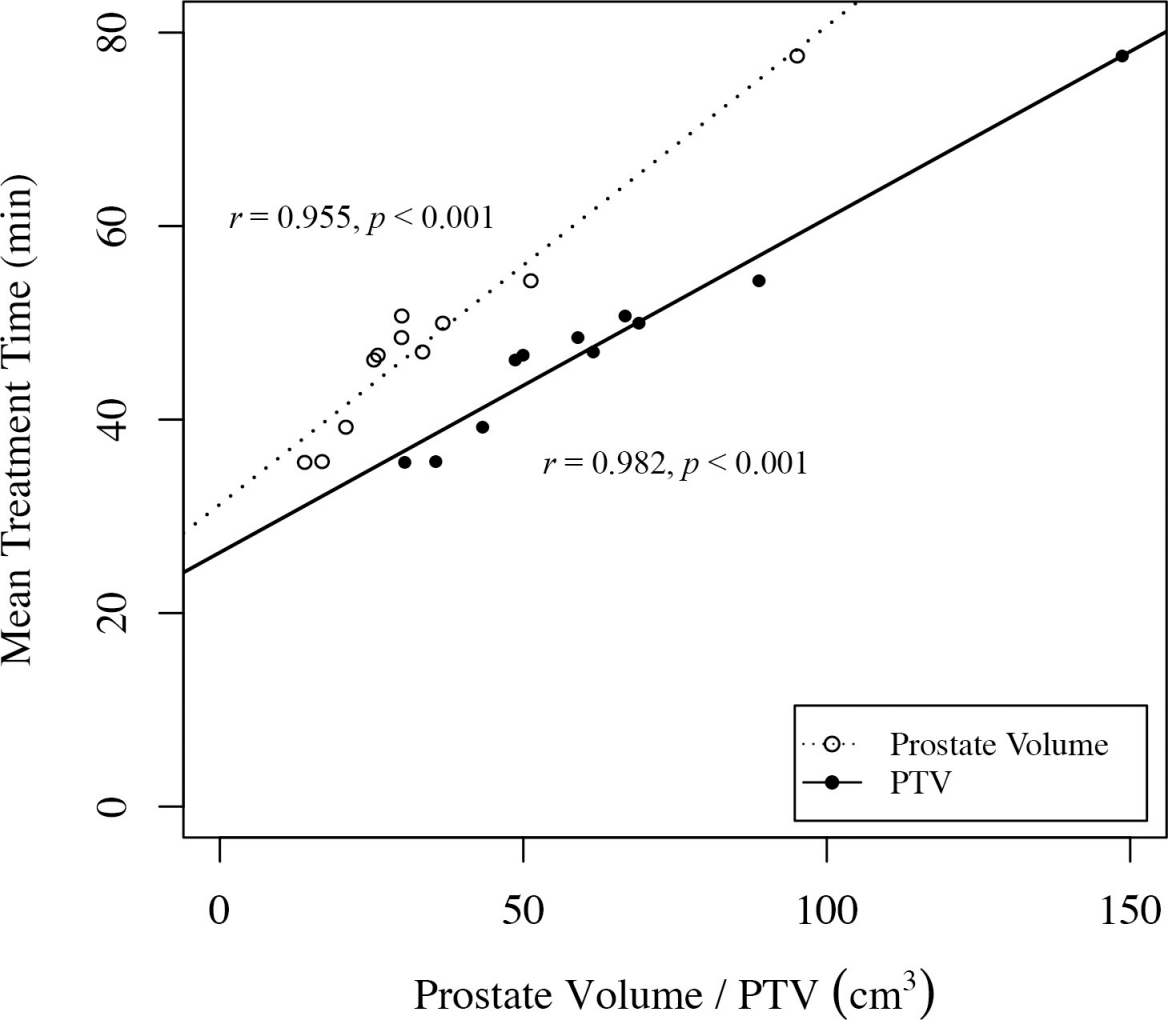
Graph of mean treatment time against prostate volume and PTV. Spearman’s correlation coefficients (*r*) and *p*-values were calculated using R software (version 3.5.0, R Foundation for Statistical Computing, Vienna, Austria).

One patient had larger penalty values for all plans than any other patient. The mean penalty value of the plans for this patient was 1.93 ± 0.30, while that for the patient with the second largest mean penalty was 0.83 ± 0.68. This is because the patient with large penalty values had a very large prostate volume (95.14 cm^3^), and hence PTV (148.70 cm^3^), in comparison with the other patients. We expected that larger prostate volumes and PTVs would require longer treatment times because more beams would be needed to achieve uniform irradiation of large targets. This was supported by our findings. A graph of the mean treatment time of plans plotted against the prostate volume and PTV of each patient confirmed a proportional relationship (Fig 4): a large prostate volume and PTV lead in increased treatment times, and consequently, in increased penalty values. Moreover, another research indicated that patients with large prostate volumes (>50 cm^3^) are more likely to develop genitourinary and gastrointestinal toxicities [34]. We therefore concluded that considerable care must be taken when designing plans for patients with large prostate volumes.

The main limitation of this research was the small sample size. This was primarily because a recent upgrade of MultiPlan to version 5.2.1 rendered it impossible to increase the sample beyond 11 patients in our cohort. A larger sample might have allowed accurate identification of any trends and would have helped generalize the results to other populations. However, the small sample size did allow us to examine individual results in much greater detail. For instance, although the 25/50 mm collimator combination was optimal for about half of the patients (Table 4), the combination produced a plan with a relatively large penalty in one case (penalty value = 1.69), indicating that it might not be suitable for use in all cases. In a large sample, we might have missed this finding. The small sample size also means that the accuracy of the superior-group analysis was less than that of the heat map analysis because few plans were included (102 and 11 plans in groups A and B, respectively) compared with the heat maps (all 220 plans). Another limitation was the impact of the treatment time on the objective function (Eq 5), as previously discussed, which might have affected accuracy when modeling the quality of treatment plans. To confirm the reliability of our results, future research could follow the same procedure, but with an improved objective function and a greater sample size. Despite these limitations, we have shown that an objective function including the treatment time for evaluating plan quality, as the one proposed in this study, was useful.

## Conclusions

Long treatment times are a disadvantage of SBRT for prostate cancer when using CyberKnife. Therefore, the treatment time should be considered when evaluating plan quality. The objective function introduced in this research enables quality evaluation and can be helpful in determining optimal collimator combinations. Our results indicate that the optimal Iris collimator combination would be a 25 mm small and a 50 mm large collimator when aiming to produce plans with the shortest treatment times that retain clinically acceptable dose distributions. However, care is needed because our results also indicate that specific cases may benefit from different combinations. The 25/40 mm combination, for example, may be a potential alternative for cases with small prostate volumes.

This research has the potential to be of great clinical importance. To date, medical physicists have been creating treatment plans with different collimator combinations before deciding which plan to use for treatment. Using the optimal combinations determined in this research would reduce planning times while ensuring acceptable dose distributions and short treatment times. Additional work is needed to establish the optimal collimator combination when using more than two Iris collimator sizes so that we can improve dose uniformity to the target and further decrease the treatment time and irradiation to the OARs.
